# African-Colombian woman with preeclampsia and high-risk *APOL1* genotype: A case report

**DOI:** 10.1097/MD.0000000000040284

**Published:** 2024-11-01

**Authors:** Carlos E Duran, Juan David Gutierrez-Medina, Jacobo Triviño Arias, Lina M Sandoval-Calle, Mario Barbosa, Elena Useche, Lorena Diaz-Ordoñez, Harry Pachajoa

**Affiliations:** a Nephrology Unit, Fundación Valle del Lili, Cali, Colombia; b Departamento de Ciencias Basicas Medicas, Facultad de Salud, Universidad Icesi, Cali, Colombia; c Centro de Investigaciones Clínicas, Fundación Valle del Lili, Cali, Colombia; d Centro de Investigaciones en Anomalias Congenitas y Enfermedades Raras (CIACER), Universidad Icesi, Cali, Colombia; e Genetic Division, Fundacion Valle del Lili, Cali, Colombia.

**Keywords:** African–Colombian, *APOL1*, case report, DNA, genetics, hypertension, mitochondrial, preeclampsia

## Abstract

**Rationale:**

Preeclampsia is one of the main causes of maternal morbidity and mortality worldwide. Even though preeclampsia is the most prevalent medical complication of pregnancy, it predominantly affects Black women when compared with other ethnicities. *APOL1* G1 and G2 risk alleles are genetic risk factors for hypertension and more recently have been associated to the risk of developing preeclampsia.

**Patient concerns:**

A 17-year-old African Colombian primigravid patient from the Colombian Pacific Coast with preeclampsia, grade 1 obesity, convulsive episodes and psychomotor agitation.

**Diagnoses:**

The patient exhibited elevated blood pressure readings concomitant with 4 tonic-clonic episodes, tachycardia, Grade I edema, irregular uterine activity and recurrent convulsive episodes. A head computed tomography revealed posterior reversible encephalopathy syndrome along with cytotoxic edema. Genetic testing unveiled a high risk *APOL1* genotype (G1/G2) and a confirmed matrilineal African genetic ancestry (haplogroup L3b).

**Interventions:**

Initial management involved administration of labetalol and sodium nitroprusside infusions alongside neuroprotective management utilizing magnesium sulfate. Due to the diagnosis of eclampsia, pregnancy termination was performed via cesarean section. The additional antihypertensive therapeutic protocol with nitroprusside, labetalol, carvedilol, and diltiazem finally controlled the hypertensive crisis.

**Outcomes:**

Discharge was provided with family planning via subdermal implant contraception and established antihypertensive management.

**Lessons:**

This is the first Latin American report of an underage patient with a hypertensive crisis of pregnancy associated with a G1/G2 high risk genotype and a verified matrilineal genetic ancestry represented by a haplogroup L3b. This case reflects the importance of considering genetic predisposition in the context of preeclampsia. A stratified approach to preeclampsia management that acknowledges genetic factors harbors the potential to significantly diminish the maternal morbidity and mortality entwined with this condition.

## 1. Introduction

Hypertensive disorders of pregnancy (HDP) are a set of complications that vary in severity and include chronic hypertension, gestational hypertension, chronic hypertension with superimposed pre-eclampsia and preeclampsia-eclampsia.^[1]^ The American College of Obstetricians and Gynecologists defines preeclampsia (PE) as new onset hypertension of ≥ 140 mmHg systolic or ≥ 90 mmHg diastolic along with proteinuria or other end organ dysfunction, in previously normotensive women with more than 20 weeks of gestation.^[2]^ PE is one of the main causes of maternal morbidity and mortality, and is associated with preterm birth, intrauterine growth restriction and perinatal death.^[3]^ PE is the most prevalent medical complication of pregnancy affecting 3% to 8% of women in developed countries and causing about 50,000 deaths worldwide due to complications.^[4]^ In Colombia, a low-middle income country, gestational hypertension and PE account for 19% of maternal deaths.^[5]^ Hypertensive disorders represent a public health problem in our country by being the leading cause of maternal morbidity and mortality with 27.8 cases per 1000 births and a mortality rate of 0.85 per 100,000 births, according to a 2020 report of the National Institute of Health.

PE predominantly affects Black women, when compared with other ethnicities.^[6]^ After controlling for socioeconomic factors, African American pregnant women have a 3-fold risk of mortality compared to White women.^[7,8]^ Colombia has the second largest population of African descendants in Latin America, with approximately 4,671,160 self-recognized individuals that live predominantly in the southwestern territory, where this population reaches about 33%.^[9]^ Compared to other ethnic or racial groups, people of African descent have a higher incidence of hypertension and related comorbidities,^[10]^ in part due to *APOL1* genetic variants that increase the likelihood of hypertension and kidney diseases in people of African descent.

It is estimated that 12 to 14% of African Americans carry 2 *APOL1* gene variants termed G1 and G2.^[11]^ Because these *APOL1* alleles confer resistance to African trypanosomiasis, the G1 and G2 variants are present only in populations of recent African heritage due to positive selective pressure.^[12]^ However, this protection arose at the expense of a greater risk of a spectrum of kidney diseases and a faster progression to hypertension in homozygotes and compound heterozygotes with 2 copies of the *APOL1* G1 and G2 risk alleles termed high-risk genotypes (G1/G1, G2/G2 and G1/G2).^[13]^ Several data support a role for *APOL1* genotype in the development of preeclampsia. Serum circulating *APOL1* is one of the differential peptides that can discriminate between PE patients and healthy pregnant controls.^[14]^ Furthermore, maternal *APOL1* G1 risk allele was significantly associated with early-onset PE development (OR 2.2, *P* = .03).^[15]^ Here we describe the first Latin-American case of a young patient with clinical characteristics compatible with PE and a genetically confirmed African ancestry and *APOL1* G1/G2 high risk genotype, which highlights the importance of considering the genetic background in this pathology.

## 2. Case presentation

### 2.1. Initial presentation

The patient was a 17-year-old primigravid patient at 32.1 weeks of gestation, hailing from Bajo Baudó on the Colombian Pacific Coast, referred from a primary care center with a medical history notable for Grade I obesity. The patient exhibited elevated blood pressure readings concomitant with 4 tonic-clonic episodes without recovery in between, meeting the definition of status epilepticus. Initial management involved exclusive administration of Labetalol and Midazolam, as the initiation of magnesium sulfate therapy was impeded by the patient’s vascular access withdrawal.

The patient presented to our emergency department in fair general condition, agitated and combative. She had blood pressure within the hypertensive crisis range, tachycardia, and normal oxygen saturation levels with a non-rebreather mask. Physical examination revealed Grade I edema, absence of neurological focalization, and irregular uterine activity. Ultrasound examination indicated a singleton fetus with signs of vitality and no evidence of fetal distress.

In response to the clinical presentation described, the patient was transferred to the resuscitation room, where anti-hypertensive treatment was initiated with a combination of labetalol and sodium nitroprusside infusions alongside neuroprotective management utilizing magnesium sulfate. However, given recurrent convulsive episodes and psychomotor agitation, Propofol was additionally administered as a therapeutic measure.

### 2.2. Clinical progression

**Figure 1.**
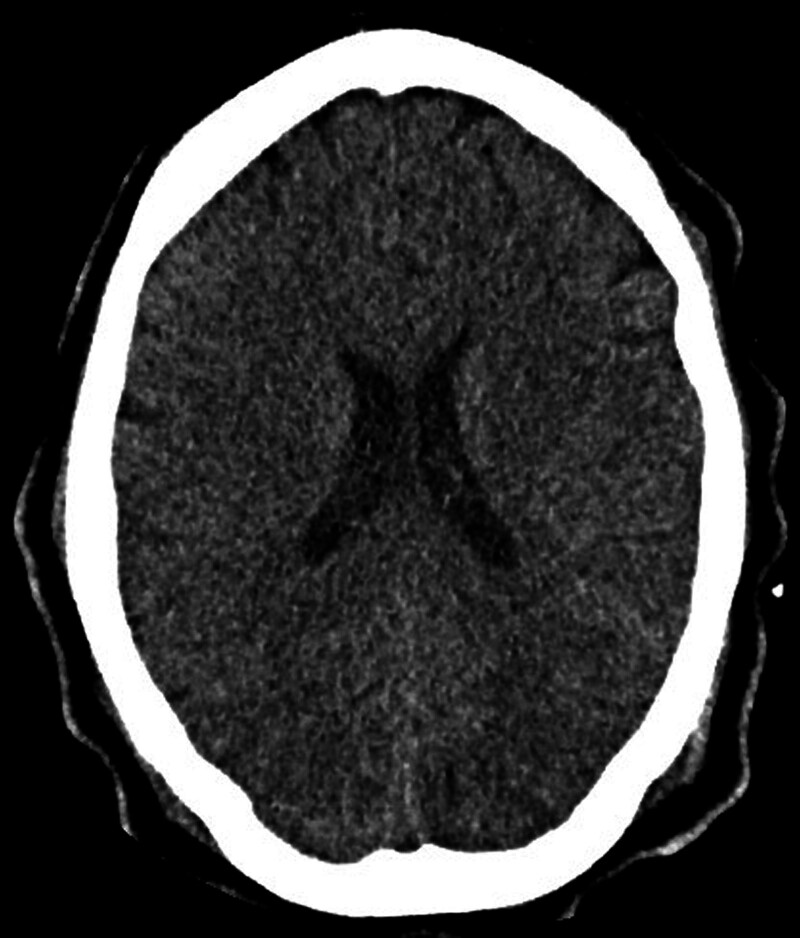
Non-contrast cranial CT after status epilepticus. CT = computed tomography.

In response to the status epilepticus and severe arterial hypertension, a head computed tomography was ordered, revealing findings consistent with posterior reversible encephalopathy syndrome along with cytotoxic edema (Fig. 1). The obstetrics and gynecology services jointly evaluated the patient. Given the diagnosis of eclampsia and the patient’s overall condition, they determined the necessity of terminating the pregnancy via a high-route approach. An emergency cesarean section was performed without intraoperative complications.

In the surgical procedure, a live female newborn was obtained, preterm of 33 weeks by Ballard, with adequate weight for gestational age. Initially, the neonate presented generalized hypotonia, bradycardia, and no respiratory effort. The neonate was managed by the neonatology service in the neonatal intensive care unit and was discharged with adequate clinical progress after 9 days of hospitalization.

In the immediate postoperative period, the mother was transferred to the intensive care unit, where she required sedation, analgesia, and orotracheal intubation, alongside anti-hypertensive therapy using a sodium nitroprusside infusion. Laboratory assessments revealed mild leukocytosis, elevated LDH levels, and maintained renal and hepatic function. Despite a favorable recovery and successful extubation, appropriate blood pressure management was challenging. An additional antihypertensive therapeutic protocol was implemented that included nitroprusside, labetalol, carvedilol, and diltiazem, finally achieving good antihypertensive control.

**Figure 2.**
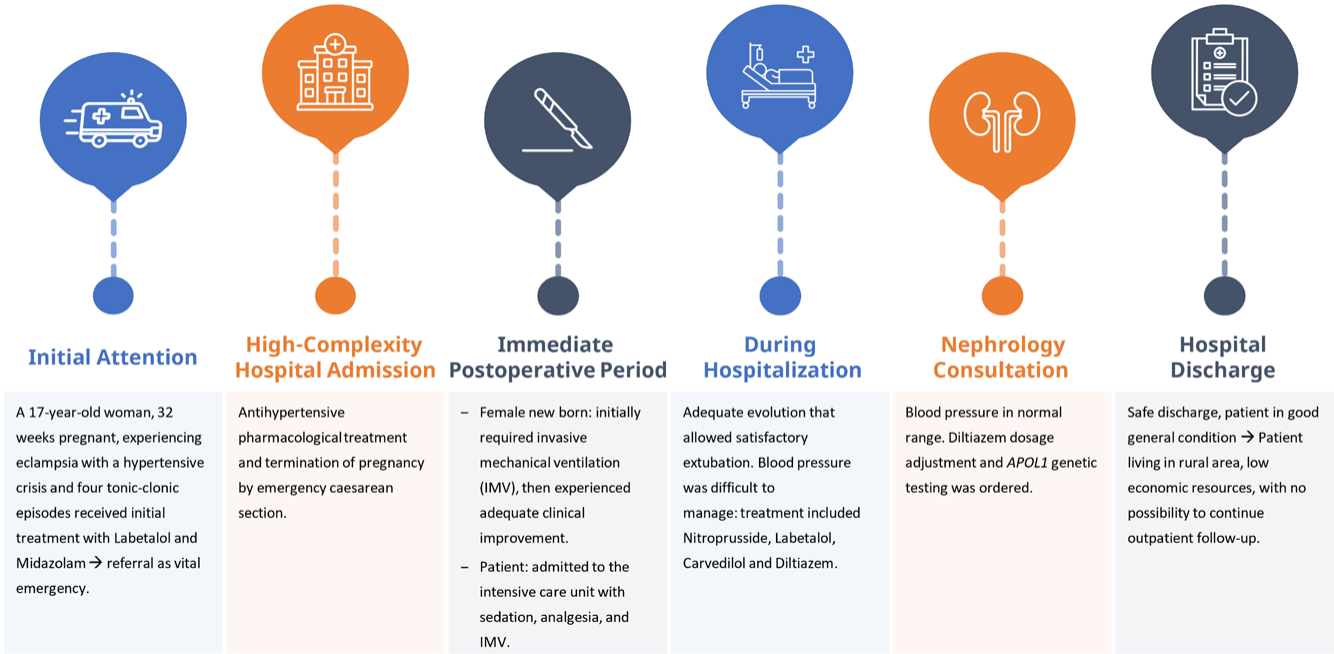
Timeline of clinical events. IMV = invasive mechanical ventilation.

Given the complexity of the case and the persistent severe arterial hypertension in a young Afro-Colombian woman with no significant medical history, consultation with the nephrology service was deemed essential. Consequently, it was performed genetic testing for *APOL1* during her hospitalization. After a 10-day hospital stay marked by notable clinical improvement, the patient had satisfactory progress and was discharged. Outpatient follow-up was advised, including additional laboratory investigations as per the nephrology service’s guidance. Clinical events are presented in Figure 2.

### 2.3. Subsequent follow-up

An underage patient with a low level of education, without a steady partner, coming from an environment of low socioeconomic resources, residing in a rural area characterized by limited access to healthcare services. This hindered the previously planned clinical follow-up. Nevertheless, discharge was provided with family planning via subdermal implant contraception and established antihypertensive management.

### 2.4. Genetic analysis

**Figure 3.**
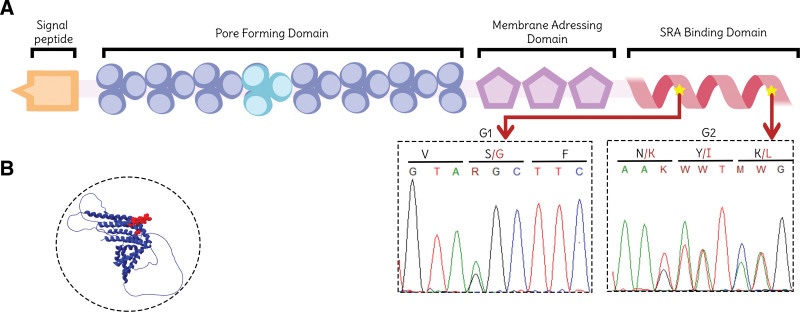
Distribution of the confirmed high-risk *APOL1* variants. (A) Domain distribution of the *APOL1* protein. The G1 and G2 variants, confirmed in our patient through Sanger sequencing, are located in the SRA Binding Domain. (B) Spatial localization of the SRA Binding Domain (red) in the *APOL1* protein structure (accession number O14791).

In order to determine the *APOL1* genotype and the matrilineal ancestry, a blood sample from the patient was collected in a 4 mL EDTA tube and DNA extraction was performed using the QIAamp DNA Mini Kit (QIAGEN, Germany) following the manufacturer’s protocol. The set of primers (amplifying a 421 bp in the exon 7 of the *APOL1* gene), amplification reaction setup, and thermocycling conditions have been described in a previous study.^[16]^ Subsequently, single-stranded Sanger sequencing was performed with the reverse primer using the BigDye® kits (Applied Biosystems, Thermo Fisher Scientific, Waltham) followed by capillary electrophoresis using the 3500 Genetic Analyzer (Thermo Fisher Scientific, Waltham). Sequence data were analyzed through MEGA X software^[17]^ using the GenBank reference sequence of *APOL1* (NG_023228.1). The patient was heterozygous for the missense variant rs73885319 that comprise the G1 allele; and heterozygous for rs71785313 which constitutes the G2 allele, reflecting a G1/G2 high risk *APOL1* genotype (Fig. 3).

Matrilineal analysis was performed by the amplification of the complete mitochondrial DNA (mtDNA) control region located between positions 16024 and 576, according to the revised Human Mitochondria Cambridge Reference Sequence.^[18]^ The primers 15876F and 639R, comprising a 1333pb amplicon, are described in a previous study.^[19]^ PCR was performed in a final volume of 20 μL, with 50 ng of DNA, 1X DreamTaq Green PCR Master Mix (Thermo Fisher Scientific, Waltham) and 0.25 μmol of each primer. The thermocycling conditions were initial denaturation at 95°C for 5 minutes; 35 cycles at 95°C for 30 seconds, 62°C for 45 seconds, and 72°C for 45 seconds; and a final extension at 72°C for 8 minutes. The same downstream workflow as with *APOL1* was applied. Sequencing of the mtDNA control region was performed with the 15876F primer and with the following primers designed by the authors: 5′-CCTATGTCGCAGTATCTGTC-3′ and 5′-CAGCACTTAAACACATCTCT-3′. Alignment was accomplished with the MEGA X software^[17]^ using the revised Human Mitochondria Cambridge Reference Sequence (NC_012920). The FASTA-formatted sequence of the mtDNA control region was submitted to MITOMASTER^[20]^ for the haplogroup determination which confirmed the African ancestry of the patient: haplogroup L3b.

## 3. Discussion

This clinical case highlights the challenges in managing a pregnant adolescent who developed severe complications during pregnancy. The patient, from a low-income rural area on the Colombian Pacific Coast, arrived at our institution with hypertensive crisis and eclampsia. Through multidisciplinary care, both mother and newborn achieved favorable outcomes. While limited access to healthcare prevented comprehensive outpatient follow-up, we conducted genetic testing that included *APOL1* analysis. Although this genetic information adds to our understanding of potential genetic factors in PE, we cannot draw definitive conclusions about causation from a single case. The relationship between *APOL1* variants and PE requires further research through larger, controlled studies before establishing any prognostic value for maternal outcomes.

HDP represent a global concern due to their significant incidence and serious consequences for maternal and perinatal health.^[2]^ PE has a disproportionate impact on regions with limited resources, where it significantly contributes to maternal deaths. Eclampsia represents the most severe expression of these disorders and carries a high risk of complications, including maternal hypoxia and trauma. Although residual neurological damage is uncommon, the sequelae can be significant, such as memory and cognitive function impairment, especially after recurrent seizures, which can lead to cytotoxic edema in patients like the one described.^[2,21]^

From the more than 10 risk factors for preeclampsia/eclampsia that the World Health Organization and the American College of Obstetricians and Gynecologists have established such as maternal age over 30 years, thrombophilia and severe anemia,^[21,22]^ our patient only had elevated body mass index and low educational level. The notable heterogeneity that PE exhibits across populations implies that not all cohorts share the same risk markers, and if there is overlap, their impact may vary substantially in each population context. Therefore, it is of outmost importance to broaden the understanding of specific risk factors in each population to formulate appropriate interventions that could have a positive effect on reducing maternal morbidity and mortality.^[21]^

Much of this disparity along with the risk factors that could play an important role in the clinical outcome of patients of specific population groups are usually not considered in clinical practice such as the *APOL1* variants. Even though there is no consensus in the pathophysiological ways that the G1 and G2 alleles may alter the biology of *APOL1*, one of the most widely accepted proposed mechanism of *APOL1*-mediated injury centers on its apparent G1 and G2 gain-of-function characteristic of forming pH-dependent ion-selective pores in the celullar unilamellar vesicular membranes, leading to osmotic imbalance and cell swelling.^[23]^

Underlying causes of PE are believed to be multifactorial, granting a role for genetic factors and hereditable predisposition. Genome wide association studies haven’t linked *APOL1* risk alleles to PE, however, considering that *APOL1* risk alleles are exclusively present in African-derived chromosomes, the exclusion of individuals of African ancestry from these genome wide association studies could be a limiting factor for the examination of the *APOL1* genetic influence.^[24]^ Hence, a role for *APOL1* in PE remains plausible. Nakimuli et al^[25]^ estimated that the prevalence of PE is higher in women descended from inhabitants from Sub-Saharan Africa than any other racial group. *APOL1* high-risk genotypes have been associated with hypertensive phenotypes, for example, Nadkarni et al^[13]^ identified an association between the *APOL1* risk alleles and higher systolic blood pressure and earlier onset of hypertension. Furthermore, placental tissue has one of the highest levels of both *APOL1* mRNA and protein expression, adding to the circulating antibodies against *APOL1* which have been found in women with PE.^[26,27]^

*APOL1* placental role is further supported by in vivo experiments in transgenic models. In a novel and unexpected finding, transgenic pregnant G2-genotype mice developed a preeclampsia/eclampsia phenotype with pregnancy-induced hypertension, proteinuria, seizures, fetal/neonatal deaths, and small litter sizes.^[28]^ Moreover, this study arose a new perspective towards the connection between *APOL1* and PE: the occurrence of the preeclampsia/eclampsia phenotype was also related to the *APOL1* genotype of the pup. In Black women, fetal *APOL1* high-risk genotype was associated with PE with odds ratios of up to 1.92 (95% CI: 1.05–3.49).^[29]^ Miller et al^[24]^ reported that infant *APOL1* genotype was significantly associated with PE in a dominant inheritance pattern with odds ratio of 1.41 (*P* = .029, 95% CI: 1.037–1.926). Importantly, Hong et al^[11]^ concluded that it was not exclusively the fetal *APOL1* genotype but rather the *APOL1* maternal-fetal genotype discordance that was associated with a 2.6-fold higher risk of PE in African Americans. Altogether, these findings suggest that both maternal and fetal *APOL1* genotypes potentially contribute to the pathogenesis of PE.

With an estimate of 35% of African Americans carrying one *APOL1* risk allele^[12,30]^ and with an Afro Colombian population of nearly 5 million people, approximately more than a million and a half self-recognized individuals carry one *APOL1* risk allele in Colombia, without counting people with African genetic ancestry that doesn’t self-recognize as Black. Duran et al^[31]^ have started to map the *APOL1* landscape in our country, revealing the relevant presence of the risk alleles in Colombia: from 102 Afro-descendant patients with end-stage renal disease, 37% of patients had *APOL1* high-risk genotype and more than 60 individuals had at least 1 risk allele.

G1 and G2 alleles originated specifically in West Africa, the region where most of the African slaves were taken from and brought to Colombia because of the colonization of the American continent.^[32,33]^ This forced migration, which changed the original Colombian gene pool, can be reconstructed through the analysis of the human mtDNA. Our patient is a young Afro Colombian woman from Chocó department, located along Colombia’s Pacific coast, where slaves were brought to work in the region’s gold mines.^[33]^ The National Administrative Department of Statistics reported that more than 80% of the population of Chocó self-identified as Afro Colombian. Our patient’s mtDNA reflected the variants corresponding to the haplogroup L3, one of the most geographically diversified sub-Saharan haplogroups and the most common in African Americans, which belongs to one of the oldest haplogroups that originated in Africa.^[34,35]^ An African mtDNA haplogroup was expected as the G1 and G2 alleles are found only in people with recent west sub-Saharan African ancestry.^[36]^

## 4. Limitations and strengths of the study

Although we thoroughly described the first case of severe PE and a genetically confirmed G1/G2 high risk *APOL1* genotype reported in Colombia, we were unable to confirm the infant’s *APOL1* genotype which, in light of new studies, seems crucial to the PE genetic context. Our patient came from a remote rural area of the Colombian Pacific Coast, which deemed subsequent follow up and sampling difficult. Finally, we were not able to measure the serum circulating *APOL1* and antibodies against *APOL1* in our patient and compare them to the levels of a healthy control.

## 5. Conclusion

This is the first case report in Latin America of PE in a young underage African-Colombian woman with a G1/G2 high risk *APOL1* genotype. The pressing need for large-scale studies targeting this delineated at-risk population is acknowledged. The comprehensive identification and characterization of genetic and ethnic factors linked with PE and pregnancy hypertensive disorders at large constitute pivotal strides toward enhancing diagnostic and prognostic capacities. While a specific treatment tailored to address the genetic predisposition identified in this case is currently absent, awareness of these factors could facilitate individualized, targeted prenatal and close monitoring, potentially mitigating the risk of severe complications. This stratified approach to PE management harbors the potential to significantly diminish the maternal morbidity and mortality entwined with this condition.

## Author contributions

**Conceptualization:** Carlos E Duran, Mario Barbosa, Elena Useche.

**Data curation:** Juan David Gutierrez-Medina.

**Investigation:** Juan David Gutierrez-Medina.

**Methodology:** Juan David Gutierrez-Medina, Jacobo Triviño Arias, Mario Barbosa, Lorena Diaz-Ordoñez, Harry Pachajoa.

**Project administration:** Carlos E Duran, Mario Barbosa, Elena Useche.

**Resources:** Elena Useche, Harry Pachajoa.

**Supervision:** Carlos E Duran, Mario Barbosa, Harry Pachajoa.

**Visualization:** Lina M Sandoval-Calle, Lorena Diaz-Ordoñez.

**Writing – original draft:** Juan David Gutierrez-Medina, Jacobo Triviño Arias, Lina M Sandoval-Calle.

**Writing – review & editing:** Carlos E Duran, Lorena Diaz-Ordoñez, Harry Pachajoa.
